# Effect of Boswellia serrata supplementation on blood lipid, hepatic enzymes and fructosamine levels in type2 diabetic patients

**DOI:** 10.1186/2251-6581-13-29

**Published:** 2014-02-04

**Authors:** Akram Ahangarpour, Hamid Heidari, Ramezani Ali Akbari Fatemeh, Mostafa Pakmehr, Hajeye Shahbazian, Iraj Ahmadi, Zahra Mombeini, Babadi Hajani Mehrangiz

**Affiliations:** 1 Diabetes Research Center, Health research institute and Department of Physiology, School of Medicine, (Golestan Street), Jundishapur University of Medical Sciences, Ahvaz 61335-189, Iran; 2 Department of Physiology, School of Medicine, (Golestan Street), Jundishapur University of Medical Sciences, Ahvaz 61335-189, Iran; 3 Department of Physiology, School of Medicine, (Saheli Street), Qom University of Medical Sciences, Qom 3713649373, Iran; 4 Physiology Research Center, Dept of Physiology, School of Medicine, (Golestan Street), Member of Student Research Committee of Ahvaz Jundishapur University of Medical Sciences, Ahvaz 61335-189, Iran; 5 Student Research Committee of Ahvaz Jundishapur University of Medical Science, School of Medicine, (Golestan Street), Ahvaz 61335-189, Iran; 6 Diabetes Research Center, Health research institute, School of Medicine, (Golestan Street), Jundishapur Medical Sciences University of Ahvaz, Ahvaz 61335-189, Iran

**Keywords:** Boswellia serrata, Fructosamine, Lipid profiles, Diabetes

## Abstract

**Background:**

Type 2 diabetes is an endocrine disorder that affects a large percentage of patients. High blood glucose causes fatty deposits in the liver which is likely to increase in SGOT and SGPT activities. Significant increase in SGOT/SGPT and low HDL levels is observed in patients with diabetes. Serum fructosamine concentration reflects the degree of blood glucose control in diabetic patients. This study was aimed to investigate the antidiabetic, hypolipidemic and hepatoprotective effects of supplementation of *Boswellia serrata* in type2 diabetic patients.

**Methods:**

60 type 2 diabetic patients from both sexes (30 males and 30 females) were dedicated to the control and intervention groups (30 subjects per group). Boswellia serrata gum resin in amount of 900 mg daily for 6 weeks were orally administered (as three 300 mg doses) in intervention group and the control group did not receive anything. Blood samples were taken at the beginning of the study and after 6 weeks. Blood levels of fructosamine, lipid profiles as well as hepatic enzyme in type 2 diabetic patients were measured.

**Results:**

Treatment of diabetic patient with Boswellia serrata was caused to significant increase in blood HDL levels as well as a remarkable decrease in cholesterol, LDL, fructosamine (p < 0.05) SGPT and SGOT levels after 6 weeks (p < 0.01). In spite of reduction of serum triglyceride, VLDL levels in intervention group, we did not detect a significant difference after 6 weeks.

**Conclusion:**

This study showed that Boswellia serrata supplementation can be beneficial in controlling blood parameters in patients with type 2 diabetes. Therefore, its use can be useful in patients with medicines.

## Introduction

Type 2 diabetes is one of the most serious endocrine disorders in worldwide [1]. It has predicted that the diabetes prevalence among the people, would reach to 552 million populations in year 2030 [2]. resistance to insulin as well as increase in generation of reactive oxygen species (ROS) and oxidative stress which result in destruction of insulin producing β-cells in pancreatic tissue, have critical roles in mechanism of induction of diabetes [3,4]. Chronic complications of diabetes are directly related to hyperglycemic conditions in the blood [5] and on the other hand the control of blood glucose in diabetic patients is poor in Iran like many other countries [6]. At the present, the current methods for cure of diabetes include exercise, diet, and the use of oral hypoglycemic agents as well as insulin therapy. However, chemical antidiabetic drugs such as biguanides and sulphonylureas have different side effects which include hypoglycemia, hepatotoxicity, and hypercoagulability [7]. In the recent years, there has been a tendency to use of medicinal plants because of the lower side effects and variety of effective compounds in plants, and especially the recommends by the World Health Organization (WHO) to use of medicinal plants [8]. *Boswellia serrata* is a plant species of Burseraceae family which grows in different region of India [9]. The main component of Boswellia serrata gum is Boswellic acids which are grups of pentacyclic terpenoids [10]. This gum has been used for many years in traditional medicine of India, china and Greek in treatment of respiratory disorders, gastric diseases and joint inflammation [9]. Its adverse effects in human are very low and ignorable and there has not been any report about serious interference of it with other drugs [11]. Different investigators have indicated to hypoglycemic effects of *Boswellia*[12]. In an animal study, a plant formulation of contain *Boswellia*, caused to beneficial effects in blood glucose reduction of STZ induced diabetic rats which its effect is comparable with fenformine [13]. In several animal studies, it is attributed to the antioxidant properties of Boswellia serrata extract [14]. In study of Pandey et al. has been shown that Boswellia serrata gum resins extract caused to reduce serum cholesterol and increase of HDL in rats [15]. Our previous study showed that 6 weeks supplementation of Boswellia serrata to type 2 diabetic patients, resulted in remarkably decrease in fasting blood glucose and increase in insulin level [16]. However, as far as we are aware, no studies have been reported on hypolipidemic effects of Boswellia serrata and its effect on SGPT level in human, the present study was performed to evaluate the effect of supplementation of Boswellia serrata on these factors among type2 diabetic patients.

## Materials and methods

The present study as clinical trial was performed in Endocrinology and Diabetes clinic of Ahvaz Jundishapur University of Medical Sciences in Iran, in 2012 (IRCT201112258515N1). The research was approved by Medical Ethical Committee of the Ahvaz University by the identification code of (eth299).

The number of samples was obtained according to the following formula $n=\frac{2{\left(Z_{1-\frac{a}{2}}+Z_{1-\frac{a}{2}}\right)}^{2}s^{2}}{\Delta^{2}}$ with consideration of α = %/05 and test power of %90 [17].

For perform of this investigation, an entry and exit criteria was considered for all participant subjects Inclusion criteria to study were 1) type 2 diabetic individuals suffering over 4 years. 2) Fasting blood glucose in range of 140–250 mg/dl 3) age range between 30–48 years. Exclusion criteria were those patients that have Hepatic cirrhosis, Chronic Kidney Disease (CKD), active Proliferative Diabetic Retinopathy (PDR), Congestive Heart Failure (CHF), Myocardial Infarction (MI) in recent 6 month, pregnant and lactating women [18]. After elucidation the aim of present investigation, 60 patients were declared their satisfaction to participate in this study and so written consent was taken from all of them. Then all individuals were randomly divided into two groups; test and control groups (30 in each group). Test individuals group received solid form of Boswellia serrata gum resin 900 milligram daily which were divided in three 300 mg doses for 6 weeks and control group have not received anything. Boswellia serrata was bought from the reliable local markets of Ahvaz city. Boswellia serrata is maintained in herbarium section of department of Botany of Ahvaz Jundishapur University and was scientifically confirmed by this department. It should be mentioned that all participant in this research have alone used oral hypoglycemic agents include GIyburide, Acarbose, Metformin and …, which didn’t changed in the type and dose of drugs during the study period. During the study, weekly telephone contacts were established with the patients and they reply the questions about any side effects such as nausea and reflux and serious drug interactions with other drugs. Also they were recommended in association with regular consumption of Boswellia serrata gum resin. Patients had their routine diet and physical activity levels until the end of the study. Demographic information's including weight, body mass index (BMI), Systolic and Diastolic pressure were measured at the beginning and end of the study. At the beginning of the study and after 6 weeks, venous blood samples were taken from all patients and were centrifuged at 3500 rpm for about 20 min for obtaining serum. Serum could be kept in -70°C refrigerator until evaluation. After serum separation, some biochemical factors include: Triglycerides, Total cholesterol, HDL, SGPT, SGOT were assessed in method of calorimetric enzymatic using commercial kits (pars azmoon, Iran), and Fructosamine (using Diazyme kit, USA). Very low-density lipoprotein cholesterol (VLDL) equals one-fifth of Triglycerides amount. For calculation the content of low density lipoprotein cholesterol (LDL), the sum of HDL and VLDL amount is subtracted from total cholesterol content and the remainder is LDL.

Data are expressed by SPSS as mean ± SEM. For this process, we used Paired *t*-test method for assessment blood parameter of before and after consumption of Boswellia serrata. Also we used the method of independent *t*-test for data analysis between two groups. P < 0.05 was considered as statistically significant difference.

## Results

In this study, 50 percent of participants were male and 50 percent were female. The average of age in Boswellia group and control group was between 30–48 years. In the beginning of study, there were not remarkable differences in means of measured factors in body and blood between the Boswellia and control groups (Table 1). After 6 weeks consuming of Boswellia, we were observed significant increase in blood HDL level and remarkable decrease in blood cholesterol, LDL, fructosamine (p < 0.05) SGPT (p < 0.001) and SGOT (p < 0.01) levels of patients. There were no significant difference in serum triglyceride and VLDL levels of patients (Table 2). In control group, HDL level was significantly increased after 6 weeks (p < 0.01). We have not observed other significant changes in blood variables at the end of study in control group (Table 3). Comparison between blood parameters of control and Boswellia groups after 6 weeks, showed significant decrease in the levels of VLDL and TG (p < 0.05) as well as total cholesterol and SGOT levels (p < 0.01) in Boswelia group rather than control group (Table 4). During the study, have not observed any side effects such as nausea and reflux, . . . and there were no reports about serious drug interactions with other drugs.

**Table 1:** The comparison of means of demographic informations and biochemical index of basal between groups

**Variables**	**Control group**	**Boswellia group**	***P*** **value**
Weight (Kg)	71 ± 3.3	68 ± 4.09	0.53
BMI (m^2^/Kg)	26.87 ± 4.1	25.93 ± 1.2	0.68
Systolic pressure (mmHg)	120.52 ± 1.3	118.88 ± 4.2	0.76
Diastolic pressure (mmHg)	76.31 ± 2.19	75.55 ± 2.9	0.84
HDL (mg/dl)	32.37 ± 2.42	35.07 ± 2.33	0.428
LDL (mg/dl)	103.59 ± 6.02	103.87 ± 6.01	0.975
VLDL (mg/dl)	38.77 ± 5.2	29.14 ± 4.22	0.149
TG (mg/dl)	194.33 ± 26.06	145.07 ± 21.07	0.144
Total Cholesterol (mg/dl)	174.79 ± 5.03	168.03 ± 7.48	0.458
SGPT (IU/L)	35 ± 4.64	32.64 ± 2.75	0.654
SGOT (IU/L)	29.107 ± 3.13	31.54 ± 3	0.58
Fructosamine (μM/L)	407.73 ± 24.39	405.78 ± 19.03	0.906

**Table 2:** The comparison of means of biochemical index in baseline and after intervention in Boswellia group

**Variables**	**Baseline**	**End 6 weeks**	***P*** **value**
HDL (mg/dl)	35.07 ± 2.33	40.39 ± 1.88	<0.05
LDL(mg/dl)	103.87 ± 6.01	88.39 ± 6.7	<0.05
VLDL (mg/dl)	29.14 ± 4.22	25.08 ± 2.36	>0.05
TG(mg/dl)	145.07 ± 21.07	129.35 ± 11.87	>0.05
Total Cholesterol(mg/dl)	168.03 ± 7.48	153.82 ± 5.89	<0.05
SGPT (IU/L)	32.64 ± 2.75	23.36 ± 1.78	<0.001
SGOT (IU/L)	29.107 ± 3.13	18.75 ± 2.12	<0.01
Fructosamine(μM/L)	405.78 ± 19.03	383.31 ± 16.41	<0.05

**Table 3:** The comparison of means of baseline and after 6 weeks biochemical index in control group

**Variables**	**Baseline**	**End 6 weeks**	***P*** **value**
HDL (mg/dl)	32.37 ± 2.42	41.66 ± 2.54	<0.01
LDL(mg/dl)	103.59 ± 6.02	110.07 ± 9.11	>0.05
VLDL (mg/dl)	38.87 ± 5.2	34.37 ± 3.34	>0.05
TG (mg/dl)	194.33 ± 26.03	171.95 ± 16.72	>0.05
Total Cholesterol (mg/dl)	174.79 ± 5.03	183.83 ± 9.42	>0.05
SGPT (IU/L)	35 ± 4.64	30.42 ± 3.2	>0.05
SGOT (IU/L)	31.54 ± 3	28.79 ± 3.18	>0.05
Fructosamine(μM/L)	407.73 ± 24.86	398.95 ± 18.82	>0.05

**Table 4:** The comparison of means of demographic information and biochemical index after 6 weeks between groups

**Variables**	**Control group**	**Boswellia group**	***P*** **value**
HDL (mg/dl)	41.66 ± 2.54	40.39 ± 1.88	>0.05
LDL(mg/dl)	110.07 ± 9.11	88.39 ± 6.7	>0.05
VLDL(mg/dl)	34.37 ± 3.34	25.08 ± 2.36	<0.05
TG (mg/dl)	171.95 ± 16.72	129.35 ± 11.87	<0.05
Total Cholesterol (mg/dl)	183.83 ± 9.42	153.82 ± 5.89	<0.01
SGPT (IU/L)	30.42 ± 3.2	23.36 ± 1.78	>0.05
SGOT (IU/L)	28.79 ± 3.18	18.75 ± 2.12	<0.01
Fructosamine(μM/L)	398.95 ± 18.82	383.31 ± 16.41	>0.05

## Discussion

In spite of several treatment options for type 2 diabetes diseases, and on the other hand, lack of definitive cure for it, more investigations are needed for treatment this disease [19]. This study evaluates the efficiency of long-term consumption of Boswellia serrata on glycaemic and lipid profiles in patients with type 2 diabetes. Our study indicates that supplementation of *Boswellia serrata* in three 300 mg doses daily for 6 weeks, significantly improves HDL, LDL and total cholesterol levels and serum SGPT, SGOT in type2 diabetic patients. Also in the present study, supplementation of diabetic patients with Boswellia serrata is reduced fructosamine level. Because the concentration of glucose is normally used to detect blood glucose control in diabetic patients, it seems that fructosamine is more useful why that used to show the decline and improve diabetic conditions over a period of several days or weeks [20]. Fructosamine is a measurement putative of glycosylated proteins and it has been suggested to measure blood value as a method for evaluating diabetes mellitus [21]. According to Elgawish study, those agents that have free radical scavenging or antioxidant properties, may help prevent oxidative reactions associated with protein glycosylation [22]. Several researchers have indicated to antioxidant effect of different membrane of *Boswellia* species [23,24]. On the other hand, a decrease in blood glucose levels may have also contributed to decreased levels of glycated proteins in diabetic patients' supplementated with Boswellia serrata. This finding is in accordance with our previous study which showed that *Boswellia serrata* supplementation in diabetic patients caused to significant decrease in fasting blood glucose level [17]. Also Awadi et al. in an animal study showed that, a plant formulation containing Boswellia was able to reduce the blood glucose of STZ induced diabetic rats [12]. For that reason, likely, Boswellia serrata with its free radical scavenging and blood glucose lowering potential, effectively reduces the formation of glycated proteins. Safayhi et al. reported that boswellic acids (the main component of Boswellia serrata) causes to hepatoprotection against galactosamine/endotoxin-induced toxicity in mice [25]. Also Jyothi study showed that Boswellia serrata oleo-gum resin can reduce liver injury caused by chemicals. [26]. As evidenced by a reduction in SGPT and SGOT among type 2 diabetic patients supplemented by Boswellia serrata, our study demonstrate that Boswellia serrata is possess partial positive hepatoprotective effect. Pandey et al. reported that Boswellia serrata oleo gum is able to decrease the production of nitric oxide [18]. It is shown that those components that are reducing the production of NO in the liver tissue possess liver protective effects [27]. Likely Boswellia serrata, through reduction of NO generation, can protect the liver function. This research showed that Boswellia serrata supplementation has beneficial effect in augmentation of HDL and reduction of total cholesterol and LDL levels among type2 diabetic patients. In agreement with our results, Pandey et al. indicated to hypocholesterolemia effect of gum resins extract of Boswellia in rats [15]. In diabetes, insulin deficiency is result in enhancement of plasma FFA concentration because of FFA explosion from body fat stores as a result of increase in lipolysis cycle due to insulin deficiency [28]. Likely Boswellia serrata supplementation restores β-cells function for insulin secretion in diabetic patients and so they will be compensated insulin deficiency. On the other hand, Insulin helps to reduce serum lipid profiles. However in spite of distinctive improvement of serum triglyceride and VLDL levels of type2 diabetic patients after supplementation with Boswellia serrata in our study, significant difference in our study did not detect varying levels after 6 weeks. The full duration of the clinical trial, as well as the lack of precise control of diet, exercise and patient education associated with diabetes who participated in our study from other sources (except physicians) to be taken. May be grounds for non-significant differences in the factors mentioned can cause in type 2 diabetes.

## Conclusion

In conclusion, the suggested health benefits of Boswellia serrata supplementation in type2 diabetic patients could ameliorate some of biochemical factor levels. Daily consumption of 900 mg of Boswellia serrata possible depicts a safe and effective means to decrease the risk factors associated with type2 diabetic subjects. If diabetes subjects use Boswellia serrata in their food regularly, they may maintain their Fructosamine levels, hepatic enzyme activities and lipid profiles close to normal levels. However, more extensive pharmacological experiments are required.

## Competing interests

The authors declare that they have no competing interests.

## Authors’ contributions

AA Supervised and conducted the study and analyzed the data and carried out the laboratory studies reviewed the manuscript, HH carried out the laboratory studies and drafted the manuscript, FR-AA and IA help in blood sample collecting and laboratory studies, MP and ZM and BHM help in patient recruitment and preparing the proposal, HS: she is a Professor in Endocrinology which Supervised the study. All authors read and approved the final manuscript.
